# Optimizing aerosol therapy in mechanically ventilated acute respiratory distress syndrome: a delivery-fidelity framework

**DOI:** 10.3389/fmed.2026.1943623

**Published:** 2026-08-25

**Authors:** Zhao-kun Fan, Zhi-rong Zhang, Ying-ying Shen, Wei Chen, Cheng-en Li

**Affiliations:** Department of Intensive Care Unit, The First Affiliated Hospital of Zhejiang Chinese Medical University (Zhejiang Provincial Hospital of Chinese Medicine), Hangzhou, China

**Keywords:** acute respiratory distress syndrome, aerosol therapy, aerosolized antibiotics, delivery fidelity, mechanical ventilation, nebulization, nebulized prostacyclins, precision medicine

## Abstract

Aerosol therapy is frequently used in adults with acute respiratory distress syndrome (ARDS) receiving invasive mechanical ventilation, yet its clinical effects remain difficult to interpret because prescribed dose is not equivalent to lung exposure. This Mini Review examines aerosol therapy as a drug-device-circuit-patient intervention by integrating ARDS guideline context, delivery evidence, drug-class literature, and implementation data. Exposure is shaped by generator and formulation, fill and residual volume, position, humidification, ventilator flow, artificial airway, filters, inline components, and interruptions. Most technical evidence is bench-derived and does not establish ARDS-specific clinical benefit. We retain gaseous inhaled nitric oxide only as a non-aerosol physiological comparator. Nebulized prostacyclins may improve oxygenation, but patient-centered benefit remains uncertain; aerosolized antibiotics should be interpreted within infection-defined ventilator-associated pneumonia; and bronchodilators or mucoactive therapies should not be used routinely on the basis of the ARDS label alone. Optimization should seek reproducible, target-appropriate exposure while preserving lung-protective ventilation, circuit integrity, treatment continuity, filtration surveillance, and staff and environmental safety. We propose a candidate minimum delivery-reporting dataset, while emphasizing that it is not a validated exposure surrogate or clinical score. Future studies should align mechanism, delivery, phenotype, response window, safety, and patient-centered endpoints to distinguish biological failure from inadequate or interrupted exposure.

## Introduction

Aerosol therapy in mechanically ventilated ARDS merits focused review because it remains clinically attractive but incompletely interpretable. Contemporary ARDS management is anchored in supportive respiratory care, including lung-protective ventilation, appropriate positive end-expiratory pressure (PEEP), prone positioning in severe hypoxemia, and individualized respiratory-support strategies (1–3). Current guidelines do not position aerosol therapy as an established disease-modifying treatment for ARDS. The purpose of delivery optimization is therefore not to broaden indiscriminate use of inhaled drugs, but to clarify whether a selected intervention can be delivered reproducibly, monitored safely, and interpreted against an appropriate physiological or clinical endpoint.

**FIGURE 1:**
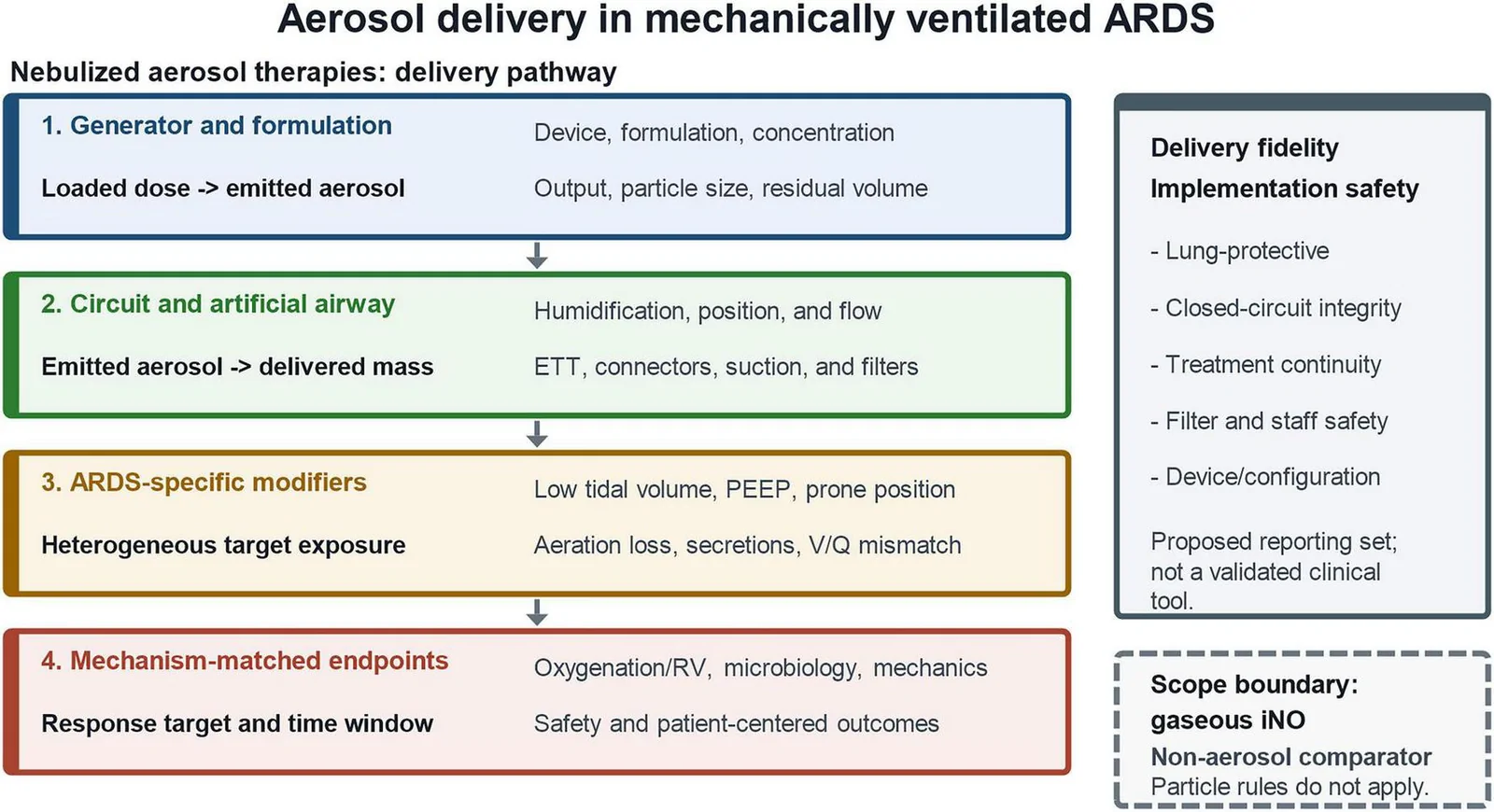
Aerosol-delivery pathway, ARDS-specific modifiers, and mechanism-matched interpretation. Four stages show how nebulized therapies move from the generator and formulation through the ventilator circuit and artificial airway to an ARDS-modified target and a mechanism-matched response. Delivered mass denotes drug mass exiting the artificial airway and should not be equated with regional deposition or target exposure. ARDS-specific factors may make target exposure heterogeneous; however, EIT characterizes regional ventilation-perfusion physiology and does not measure aerosol deposition. Delivery fidelity and implementation safety apply throughout the aerosol pathway. Gaseous iNO appears only as a non-aerosol comparator outside the particle-delivery pathway. The reporting and monitoring elements are proposed and do not constitute a validated exposure measure or clinical tool. ARDS, acute respiratory distress syndrome; EIT, electrical impedance tomography; ETT, endotracheal tube; iNO, inhaled nitric oxide; PEEP, positive end-expiratory pressure; RV, right ventricle; V/Q, ventilation-perfusion.

In mechanically ventilated ARDS, nebulized prostacyclins, aerosolized antibiotics, bronchodilators, and secretion-directed therapies address different indications. Their loaded dose must traverse a generator, humidified circuit, artificial airway, filters, connectors, suction systems, and the heterogeneous injured lung; treatment is therefore a drug-device-circuit-patient intervention (Figure 1). Inhaled nitric oxide (iNO) is delivered as a gas and is considered only as a physiological and clinical comparator; particle size, residual volume, and nebulizer position do not apply.

The strongest aerosol-delivery evidence is predominantly technical, whereas clinically relevant questions are phenotype- and endpoint-dependent. Delivery during invasive ventilation is highly variable and may be substantially lower than nominal dose (4, 5). ARDS adds regional aeration loss, ventilation-perfusion mismatch, PEEP dependence, secretions, prone positioning, and frequent airway procedures. An absent response may therefore reflect drug biology, patient selection, inadequate exposure, interruption, or an insensitive endpoint.

## Search strategy and scope

This focused Mini Review is a narrative synthesis rather than a PRISMA-style systematic review. PubMed and Web of Science Core Collection were searched through July 10, 2026, with supplementary targeted update searches and citation verification through August 1, 2026. Representative searches were the PubMed query (“acute respiratory distress syndrome”[Title/Abstract] OR ARDS[Title/Abstract]) AND (aerosol*[Title/Abstract] OR nebuliz*[Title/Abstract] OR inhaled[Title/Abstract]) and the Web of Science Core Collection Topic query (“acute respiratory distress syndrome” OR ARDS) AND (aerosol* OR nebuliz* OR inhaled). Additional targeted searches applied analogous Boolean combinations to aerosol generators and ventilator-circuit determinants; inhaled nitric oxide and prostacyclins; inhaled antibiotics, bronchodilators, and mucoactive therapies; ARDS phenotypes and regional physiology; and fugitive aerosol, filtration, and circuit integrity. Reference lists of relevant guidelines and reviews were hand-searched, and bibliographic metadata and digital object identifiers were checked against PubMed, Crossref, and publisher records.

No language or publication-status filter was applied at the database-interface stage. During narrative eligibility assessment, inclusion was restricted to English-language, peer-reviewed full-length journal articles and clinical practice guidelines, including online-ahead-of-print articles with stable bibliographic identifiers. Conference abstracts, preprints, protocols, dissertations, and non-peer-reviewed web materials were excluded from the final evidence set. We prioritized guidelines, systematic reviews and meta-analyses, clinical studies, and contemporary invasive-ventilation delivery studies; older studies were retained for foundational mechanisms, configurations not addressed by newer evidence, or evidence boundaries. Pediatric or neonatal studies, non-invasive respiratory support, and spontaneously breathing populations were excluded unless they clarified a shared delivery mechanism. One reviewer performed narrative selection, and 42 publications were retained in the final synthesis. Because no prospective screening, deduplication, or exclusion log was maintained, database-specific screening counts cannot be reconstructed; no PRISMA flow diagram or quantitative synthesis was undertaken.

The primary scope was therapies delivered as aerosols through invasive ventilator circuits in adults. Inhaled nitric oxide was retained only as a non-aerosol comparator for pulmonary-vasodilator physiology, response heterogeneity, and clinical outcomes, and was excluded from analyses of aerosol generation, particle transport, deposition, residual volume, and nebulizer position. The synthesis asks whether intended aerosol exposure can be delivered reproducibly, monitored safely, and interpreted against an appropriate endpoint in mechanically ventilated ARDS.

## Nominal dose is not lung dose: the aerosol delivery chain

Nominal dose denotes the amount prescribed or loaded; emitted dose, the amount leaving the generator; delivered or inhaled mass, the amount measured distal to the artificial airway or on a collection filter; and deposited drug mass, the amount collected at a defined downstream or anatomical surrogate. Lung exposure is the drug available at the relevant pulmonary target and is rarely measured directly in the cited bench studies. Target-appropriate exposure is an interpretive construct linking measured delivery to the intended site and mechanism. Most bench or *ex vivo* studies quantify delivery surrogates, not regional lung exposure or ARDS-specific benefit (4, 5). These terms apply to aerosol devices, not gaseous iNO.

Generator type is an upstream determinant, but its effect is inseparable from formulation, circuit, and ventilator conditions. Vibrating-mesh nebulizers (VMNs), conventional jet nebulizers (JNs), breath-enhanced jet nebulizers, and pressurized metered-dose inhalers (pMDIs) differ in driving-flow requirements, residual volume, output characteristics, formulation compatibility, and workflow (6). Formulation properties, aerodynamic particle-size distribution, fill volume, and residual volume can influence emitted and delivered dose, with effects that vary by device (6, 7). In one bench comparison, VMN inhaled mass fraction was not significantly affected by fill volume or humidification, but delivery was highly variable and incomplete device emptying occurred frequently (7). VMNs generate aerosol without adding pneumatic driving flow, yet their delivered dose remains dependent on circuit position, ventilator settings, humidification, and bias flow (6–9).

For continuous aerosol delivery, additional operational variables include infusion-pump flow rate, duty cycle, ventilator settings, total infusion time, and device integrity (10, 11). These factors have drug-class-specific implications. Uninterrupted and stable aerosol output is particularly important for continuously nebulized prostacyclins because treatment interruption may result in rapid loss of physiological effect and rebound instability (11). For aerosolized antibiotics, delivery performance must be considered together with formulation compatibility and pharmacokinetic/pharmacodynamic targets, whereas bronchodilator response should be interpreted using airway resistance, expiratory flow, and other respiratory-mechanics measures.

No nebulizer position is universally optimal. In a heated-humidifier model, placement at the humidifier inlet increased delivery, whereas increasing bias flow from 2 to 5 L/min reduced it, with device-dependent effects (9). A later multistage VMN model found that exhaled and active humidity attenuated ventilator-setting and position effects (8). An *ex vivo* porcine model favored placement 15 cm before the Y-piece but found that humidification enlarged particles and shifted deposition centrally (12); a clinical JN study and a humidified adult bench model reported different preferred locations under their tested configurations (13, 14). These findings support reporting and selecting position in the context of device, operating mode, humidification, circuit geometry, flow, and measurement endpoint.

Circuit architecture and inline components also affect distal delivery. In simulation, a filtered heat-and-moisture exchanger (HME) between generator and patient reduced delivered dose below 0.5% of nominal, whereas the tested nonfiltered HME passed more than 60% of the control dose; HME type should therefore be specified, and local circuit protocols followed (15). Stopping heated humidification did not improve delivery of three tested drugs and rapidly lowered absolute humidity, arguing against routine interruption solely to increase dose (16). Lung-protective settings should not be altered for aerosol optimization (1–3). In bench models, an inspiratory pause did not improve distal mass, lower flow increased delivery, an inline suction catheter reduced delivery, and pMDI adapter design mattered more than suction-catheter design (17, 18). These configuration-specific findings support standardized reporting, not universal bedside settings.

We propose a minimum reporting dataset covering device and formulation; fill and residual volume; circuit and position; humidification; artificial airway; ventilator settings and flow; filters and inline components; duration and interruptions; response window; and safety events. It is an unvalidated reporting construct, not an exposure surrogate, clinical instrument, or benefit measure, and requires consensus and prospective validation.

## ARDS-specific modifiers of aerosol delivery and interpretation

Acute respiratory distress syndrome does not alter the fundamental principles of aerosol transport, but it changes the clinical conditions under which delivered dose is generated and interpreted. Low-tidal-volume ventilation, appropriate positive end-expiratory pressure (PEEP), and prone positioning in severe ARDS should remain governed by lung-protective objectives rather than modified solely to increase aerosol delivery (1–3). Bench and simulation studies indicate that inspiratory flow, inspiratory timing, circuit configuration, and inline components can modify drug mass measured distal to the artificial airway under specific experimental conditions (4, 5, 17, 18). However, these findings do not establish that corresponding ventilator adjustments improve regional lung exposure or clinical outcomes in ARDS.

Electrical impedance tomography (EIT) in 50 mechanically ventilated patients demonstrated regional ventilation-perfusion mismatch associated with mortality (19). Aerosol may plausibly favor better-ventilated regions, whereas consolidated or mucus-obstructed units may be less accessible, but regional aerosol imaging has not established this relationship in ARDS. Drug mass measured at the endotracheal-tube outlet or on a collection filter is a delivery surrogate, not evidence of homogeneous deposition or target exposure. Prone positioning redistributed ventilation and perfusion in a small physiological study (20), but its effect on aerosol deposition remains unknown; patient position and treatment timing should be recorded rather than assumed to improve delivery.

At high PEEP, circuit opening causes loss of airway pressure and may promote derecruitment, supporting circuit integrity when clinically and device compatible (21). Secretions, mucus plugging, and inline suction may impair airway patency or distal delivery under specific conditions (17, 18, 22). Suctioning, bronchoscopy, recruitment maneuvers, transport, position changes, and interruptions should be recorded as co-interventions, although their independent effects on regional deposition remain uncertain. Pulmonary vascular and right-ventricular phenotypes inform vasodilator indication and response interpretation rather than aerosol efficiency (23–26).

## Drug-class evidence through a delivery lens

After delivery conditions are defined, interpretation must align drug mechanism, intended site, phenotype, response window, and endpoint. Nebulized prostacyclins, antibiotics, bronchodilators, and secretion-directed therapies therefore require distinct indications and response measures (Table 1).

**TABLE 1:** Proposed modality-specific framework for interpreting aerosol therapies, with gaseous inhaled nitric oxide included as a non-aerosol comparator.

Therapy/modality	Clinical context	Delivery/reporting elements	Response and safety monitoring	Evidence source/level and key references	Key evidence gap
Gaseous iNO (non-aerosol comparator)	Selected rescue use for refractory hypoxemia or pulmonary vascular/RV involvement; not established as routine ARDS therapy	Calibrated gas-delivery system; delivered NO concentration; inspired NO_2_ and FiO_2_; continuity, alarms, and backup delivery	Oxygenation, blood pressure/RV response, methemoglobin level and renal function; interruption, weaning, and rebound	Narrative review, physiological/observational studies, and systematic reviews (23, 25–28)	Define responder phenotypes and link short-term physiological effects to patient-centered outcomes
Nebulized prostacyclin analogues	Selected adjunctive or rescue use for refractory hypoxemia or pulmonary vascular/RV involvement	Formulation/concentration; aerosol generator; infusion rate; residual volume; circuit position; humidification; output continuity	Oxygenation and blood pressure/RV response; platelet count and clinically relevant bleeding, particularly with thrombocytopenia, coagulopathy, or concomitant antithrombotic therapy; bronchospasm; treatment interruptions, continuation criteria, and weaning	Technical review and low-certainty systematic review/meta-analysis (11, 24)	Phenotype-enriched trials linking measured delivery conditions to patient-centered outcomes
Aerosolized antibiotics	Microbiologically defined VAP in selected contexts; ARDS alone is not an indication	Compatible formulation/device; circuit conditions; treatment completion; microbiology and systemic co-therapy	Clinical and microbiological response; bronchospasm and filter burden; renal function and other drug-specific systemic adverse effects, particularly with concomitant systemic antibiotic therapy; antimicrobial stewardship	Meta-analyses, pharmacological review, guidance, and configuration-specific bench evidence (29–32, 39)	Pathogen-specific trials linking measured pulmonary exposure to clinical outcomes and safety
Bronchodilators	Objectively supported reversible airflow obstruction or bronchospasm, including COPD/asthma overlap	Consistent device/circuit configuration with interpretable pre/post respiratory mechanics	Airway resistance, expiratory flow, auto-PEEP, dyssynchrony, and airway-pressure response; avoid routine ARDS-directed use	ARDS randomized clinical trial; otherwise mechanism-based use for a coexisting airway indication (33)	Mechanism-specific trials using prespecified airway-mechanics endpoints
Secretion-directed therapies	Documented secretion retention, mucus plugging, or selected atelectatic complications	Integration with an airway-clearance plan; device/circuit conditions and suction timing documented	Airway patency, mucus plugging, atelectasis, suctioning burden, and ventilator mechanics; bronchospasm or transient obstruction	Systematic review and implementation study; efficacy remains insufficient for routine unselected use (34, 35)	Patient selection and clinically meaningful secretion-related endpoints
Aerosol delivery and reporting elements	Applies across aerosol therapies in mechanically ventilated ARDS	Device, formulation, fill/residual volume, position, humidification, artificial airway, filters, suctioning, duration, and interruptions	Delivered/inhaled mass definition, protocol adherence, circuit integrity, filter resistance, fugitive aerosol, and safety events	Bench/*ex vivo* and clinical delivery studies, reviews, and practice data (4–18, 21, 22, 36–39); author-proposed reporting construct	Prospective validation of the minimum dataset against measured exposure and clinical outcomes

This table is a proposed interpretive and reporting framework, not a validated clinical decision tool or treatment recommendation. ARDS, acute respiratory distress syndrome; COPD, chronic obstructive pulmonary disease; FiO_2_, fraction of inspired oxygen; iNO, inhaled nitric oxide; NO_2_, nitrogen dioxide; PEEP, positive end-expiratory pressure; RV, right ventricle; VAP, ventilator-associated pneumonia.

Nebulized prostacyclins belong to the aerosol framework, whereas iNO is a non-aerosol comparator. Prostacyclins may be considered as adjunctive or rescue therapy in selected severe ARDS with refractory hypoxemia or pulmonary vascular or right-ventricular involvement. They may improve oxygenation, but evidence is heterogeneous and low certainty without established patient-centered benefit (24). Delivered exposure depends on formulation, generator, circuit, humidification, output continuity, and interruption (11). Because prostacyclins inhibit platelet aggregation, safety assessment should include platelet count and clinically relevant bleeding, particularly in patients with thrombocytopenia, coagulopathy, or concomitant antithrombotic therapy; however, adverse-event reporting is inconsistent and current evidence does not quantify an excess bleeding risk (24).

Inhaled nitric oxide is delivered through a calibrated gas system and is not governed by aerosol variables. Physiological and observational studies show heterogeneous responses in ventilation-perfusion matching and right-ventricular function, whereas systematic reviews show modest or transient oxygenation improvement without consistent mortality benefit in unselected ARDS (23, 25–28). It informs phenotype and endpoint selection, not aerosol efficiency. When used, reporting should document delivered NO concentration, inspired nitrogen dioxide (NO_2_), fraction of inspired oxygen (FiO_2_), continuity, alarms and backup, methemoglobin level, renal function, hemodynamic response, and weaning or rebound; nebulized-prostacyclin reporting should instead capture aerosol-system performance and continuity.

Aerosolized antibiotics should be framed by infection-defined ventilator-associated pneumonia (VAP), not ARDS. Meta-analyses suggest possible improvement in clinical cure or microbiological eradication, but heterogeneity is substantial and mortality effects remain inconsistent (29, 30). Any local-exposure advantage depends on drug, formulation, generator, circuit, regional distribution, and measurement (31). IDSA guidance does not suggest nebulized antibiotics for difficult-to-treat resistant *Pseudomonas aeruginosa* or carbapenem-resistant *Acinetobacter baumannii* respiratory infections because trials lack consistent benefit and raise distribution and respiratory-adverse-effect concerns (32). When considered, use should be linked to microbiology and address formulation, systemic therapy, bronchospasm, delivery conditions, and stewardship. Safety assessment should include renal function and other drug-specific systemic adverse effects, particularly when inhaled and systemic antibiotics are co-administered. An earlier meta-analysis found no increased pooled risk of nephrotoxicity, whereas a recent analysis reported lower nephrotoxicity than intravenous therapy in an exploratory comparison based on three randomized trials. However, nephrotoxicity was reported in only a subset of studies, definitions and treatment regimens varied, and concomitant systemic antibiotic therapy may complicate attribution; these findings should not be interpreted as establishing the absence of drug-specific systemic toxicity (29, 30).

Bronchodilators and secretion-directed therapies further illustrate why the ARDS label alone is insufficient to define an indication. Inhaled β_2_-agonists should not be used routinely as ARDS-directed therapy because randomized evidence has not demonstrated improvement in clinically important outcomes (33). They may still be appropriate for a coexisting indication when reversible airflow obstruction or bronchospasm is supported by examination, expiratory flow, or respiratory-mechanics findings. Evidence for routine nebulized mucoactive therapy in mechanically ventilated adults remains insufficient, and implementation studies do not establish efficacy for unselected secretion clearance (34, 35). Individualized use may be considered for documented secretion retention, mucus plugging, or selected atelectatic complications, integrated with an airway-clearance plan and accompanied by monitoring for bronchospasm, transient obstruction, and changes in ventilator mechanics.

## Safety and implementation: delivery fidelity is part of efficacy

Delivery fidelity and safety are interdependent. A bench-optimal setup may be unsuitable if it opens the circuit in a PEEP-dependent patient; filtration can reduce emissions, but filter loading can raise resistance; and interruption of continuous therapy can alter response. For nebulized prostacyclins, interruption may rapidly reverse physiological effect (11). These factors affect exposure, safety, and interpretation.

An intact circuit may be prioritized when device and clinical context permit. In simulation, opening a jet nebulizer for refill eliminated PEEP and released simulated bioaerosol, whereas refilling an inline vibrating-mesh nebulizer preserved pressure without a detected particle increase above ambient (21); this demonstrates mechanisms, not clinical derecruitment or infection risk. Inline generators, adapters, and closed suction can reduce disconnections but may alter aerosol transport (17, 18). Because guidance does not establish universal superiority of one suction system, selection should reflect secretion burden, airway pressures, PEEP, local protocols, and circuit integrity (22). Access, refill, and interruption risk should be planned before high-PEEP, prone, transport, or continuous therapy.

Fugitive aerosol pathways vary with ventilatory interface, nebulizer, tidal volume, filtration, circuit opening, and refill workflow (21, 36, 37). Invasive ventilation is relatively contained only with an intact circuit and filtered exhaust; open ports, manual ventilation, and disconnections create release routes. Therapeutic aerosol and infectious bioaerosol should be distinguished although both may coexist. These simulations identify potential release pathways rather than occupational infection risk.

Real-world practice data illustrate the gap between technical recommendations and bedside delivery. In a secondary analysis of the prospective multicenter Aero-in-ICU study, 51 of 53 patients with ARDS received 1,285 aerosol sessions, with jet nebulizers accounting for 55.4% of treatments. During invasive mechanical ventilation, the aerosol generator was placed at the study-defined recommended position 15–30 cm from the Y-piece in 460 of 918 sessions (50.1%), and no expiratory-filter use was recorded (38). These findings document substantial practice variation but do not establish associations with lung exposure, physiological response, adverse events, or clinical outcomes.

In a heated and humidified bench model, repeated colistimethate delivery with a vibrating-mesh nebulizer at the Y-adapter produced more expiratory deposition and filter-pressure rise than humidifier-inlet placement over 14 treatments (39). Results are configuration-specific and do not establish clinical thresholds or harm. Surveillance during repeated or continuous therapy may include airway pressures, expiratory flow, PEEP, tidal volume, alarms, continuity, and manufacturer-specified filter replacement. Delivery fidelity encompasses output, circuit integrity, filter surveillance, continuity, and staff and environmental protection.

## Discussion

This review frames aerosol therapy as a drug-device-circuit-patient intervention. Nominal dose is not lung exposure; optimization should seek reproducible, target-appropriate exposure without compromising lung protection; and delivery fidelity and safety are inseparable because circuit opening, interruption, filter loading, and emissions alter both intervention and interpretation.

Evidence certainty differs by domain. Bench, preclinical, and selected clinical delivery studies show configuration-specific effects of generator, formulation, position, humidity, flow, airway components, and filtration (4–18), but do not establish ARDS-specific clinical benefit. EIT shows regional ventilation-perfusion heterogeneity and prone redistribution, not aerosol deposition (19, 20). Prostacyclin oxygenation benefit remains low certainty (24); iNO provides physiological context, not aerosol evidence (23, 25–28). Antibiotic evidence is mainly VAP-specific (29–32), while bronchodilators and mucoactive therapies lack support for routine syndrome-directed use (33–35).

The immediate priority is standardized reporting. The proposed dataset requires testing for completeness, feasibility, reproducibility, and association with measured exposure and outcomes. Device uptime, residual volume, waveforms, interruption logs, and filter surveillance may indicate delivery performance but are not validated exposure measures. Practice variation and filter loading support including adherence and delivery-related safety outcomes (38, 39).

Phenotype matching remains unvalidated for aerosol therapy (40–42). Prostacyclin studies could evaluate pulmonary vascular or right-ventricular dysfunction and early response while prespecifying platelet count and clinically relevant bleeding outcomes; antibiotic trials should define infection and integrate microbiology, systemic therapy, delivery, stewardship, renal function, and other drug-specific systemic adverse effects (24, 29, 30). Bronchodilator and secretion-directed studies should require objective airway indications. Position, suctioning, bronchoscopy, recruitment, transport, and interruption should be recorded as co-interventions, and responder variables tested rather than assumed.

Limitations include narrative selection, heterogeneous bench configurations, mixed clinical populations, inconsistent endpoints, and incomplete delivery reporting. Endotracheal-tube outlet or filter mass is not regional deposition or target exposure. Gaseous iNO can inform physiological rationale and endpoint selection but cannot validate an aerosol-delivery framework. The proposed dataset and phenotype priorities are testable recommendations, not standards of care.

The objective is reproducible, target-appropriate aerosol exposure in selected patients while preserving lung protection, circuit integrity, continuity, and safety. Trials must align mechanism, delivery, phenotype, response window, safety, and patient-centered endpoints to distinguish biological failure from inadequate or interrupted exposure.
